# Left Atrial Veno-Arterial Extracorporeal Membrane Oxygenation In Valvular Cardiogenic Shock

**DOI:** 10.1016/j.jscai.2025.102615

**Published:** 2025-05-01

**Authors:** Gennaro Giustino, Raef Ali Fadel, Ahmad Jabri, Jennifer Cowger, Brian O’Neill, Mir Babar Basir, Pedro Engel Gonzalez, Tiberio Frisoli, James Lee, Philippe Généreux, William W. O’Neill, Pedro A. Villablanca

**Affiliations:** a Valve and Structural Heart Disease Center, Gagnon Cardiovascular Institute, Atlantic Health System, Morristown, New Jersey; b Center for Structural Heart Disease, Henry Ford Hospital, Detroit, Michigan; c Department of Cardiovascular Medicine, Heart and Vascular Institute, Henry Ford Hospital, Detroit, Michigan; d Advanced Heart Failure and Transplant, Heart and Vascular Institute, Henry Ford Hospital, Detroit, Michigan

**Keywords:** Cardiogenic shock, extracorporeal membrane oxygenation, structural heart disease

## Abstract

**Background:**

Treatment of valvular cardiogenic shock (VCS) is challenging as the options for mechanical cardiocirculatory support are limited. Left atrial veno-arterial extracorporeal membrane oxygenation (LAVA-ECMO) is a mechanical cardiocirculatory support strategy that provides cardiocirculatory support and simultaneous left ventricular unloading, compared to traditional VA-ECMO.

**Methods:**

This is a single-center retrospective analysis of patients with VCS who underwent LAVA-ECMO between 2018 and 2023. During LAVA-ECMO, the ECMO venous cannula is placed transeptally in the LA, therefore providing active biventricular unloading.

**Results:**

A total of 18 patients who had VCS and underwent LAVA-ECMO cannulation were included. Among patients with VCS, 10 were related to the aortic valve (55.6%), 7 to the mitral valve (38.9%), and 1 to the tricuspid valve (5.6%). Four patients (22.2%) had multivalvular disease. The median age was 65 years, most were men (66.7%) and most were in Society for Cardiovascular Angiography & Interventions cardiogenic shock stage D or E (89%). LAVA-ECMO was associated with substantial improvement in hemodynamics, including lower right atrial pressure (–8 mm Hg; 95% CI, 7.0-9.5; *P* = .004), mean pulmonary artery systolic pressure (–18.5 mm Hg; 95% CI, 14.3-21.7; *P* = .026), pulmonary capillary wedge pressure (–14.5 mm Hg; 95% CI, 12.8-12.3; *P* = .003), and left ventricular end-diastolic pressure (–20.0 mm Hg; 95% CI, 16.5-21.0; *P* < .001). These effects were consistent across VCS types. There were no complications from transeptal cannulation. Survival to a transcatheter or surgical procedure was 69.1%, and survival to hospital discharge was 44.4%.

**Conclusions:**

LAVA-ECMO appears to be feasible, safe, and associated with improved hemodynamics in patients with VCS. Further research is needed to evaluate whether LAVA-ECMO as a bridge treatment strategy to intervention is beneficial in VCS.

## Introduction

Cardiogenic shock (CS) is a life-threatening condition associated with substantial morbidity and mortality.^1^ Valvular heart dysfunction (VHD)-related CS (VCS) accounts for overall 10% to 20% of causes of CS and its management remains challenging.^2^^,^^3^ Patients with VCS are usually excluded from randomized controlled trials evaluating mechanical cardiocirculatory support (MCS) devices.^3^^,^^4^ In addition, the presence of specific VHD lesions contraindicates the use of certain MCS devices.^3^^,^^4^

Veno-arterial extracorporeal membrane oxygenation (VA-ECMO) is an MCS strategy for CS that restores end-organ perfusion and consists of the drainage of deoxygenated blood via fenestrated cannulas, typically from the right atrium and vena cava into a pump and oxygenator which is then returned to the abdominal aorta.^5^ Hemodynamically, VA-ECMO restores systemic mean arterial pressure but significantly increases left ventricular (LV) afterload, which can result in increased myocardial oxygen demand, increased LV end-diastolic pressure (LVEDP), and worsening pulmonary edema.^5^ Strategies to mitigate the deleterious LV hemodynamic effects of VA-ECMO include additional pharmacological inotropic support, percutaneous LV unloading with an additional MCS device (eg, Impella or intra-aortic balloon pump [IABP]), and surgical LV venting.^6^ Left atrial VA-ECMO (LAVA-ECMO) is a strategy that provides VA-ECMO support while actively draining blood with a venous transeptal cannula from the left atrium (LA).^6^^,^^7^ Given that the blood is directly drained from the LA, pulmonary capillary wedge pressure (PCWP) and LVEDP can decrease with this approach enhancing pulmonary decongestion.^6^ In addition, as the blood is drained directly from the LA and right atrium (RA), LAVA-ECMO bypasses both right-sided and left-sided VHD lesions. Previously, the role of LAVA-ECMO in VHD-related CS has only been reported in small case series or case reports.^8^, ^9^, ^10^, ^11^, ^12^, ^13^, ^14^ In the present study we report, to the best of our knowledge, the largest series of LAVA-ECMO in patients with VCS.

## Methods

### Study design and population

This is a retrospective cohort study of consecutive patients presenting with CS who underwent LAVA-ECMO cannulation at Henry Ford Hospital (Detroit, MI) between January 2018 and September 2023. The present study included only patients presenting with VCS. Exclusion criteria included previous VA-ECMO cannulation at an outside hospital, central access cannulation, or missing data on VA-ECMO indication, procedural details, or outcomes. The diagnosis of VCS was documented concurrent with patient care using standard definitions including a systolic blood pressure <90 mm Hg; a need for vasopressors to maintain a mean arterial pressure of more than 60 mm Hg or a right heart catheterization documenting a cardiac index ≤2.2 L/min/m^2^ associated with signs of impaired end-organ perfusion. VCS was defined as the presence of an acute severe VHD or an acute on chronic VHD and was implicated as the primary etiology of CS. Patients who presented with an acute myocardial infarction–related CS and had a concomitant VHD were not included in this cohort of patients. Among patients on LAVA-ECMO support, the goal activated clotting time was >300 at the time of insertion, and the anti-Xa level was between 0.2 to 0.5 units/mL during maintenance using unfractionated heparin. The study received approval from the institutional review board, and the need for informed consent was waived.

### Data collection

Data were obtained through chart review and manual extraction from the electronic health record. Collected data included baseline demographics, comorbidities, hemodynamic data, and clinical outcomes. Data on ECMO cannulation, including indications, procedural details, and complications, were manually abstracted from procedural reports.

### Outcome measures and study definitions

Primary clinical outcomes of interest in our study were the rate of in-hospital mortality and the rates of successful LAVA-ECMO decannulation. Secondary assessments included post–LAVA-ECMO cannulation hemodynamics assessed with a right heart catheterization. Additionally, we collected safety end points including acute kidney injury (AKI), major bleeding (including retroperitoneal bleeding), vascular complications, and thrombocytopenia. Society for Cardiovascular Angiography & Interventions (SCAI) stages of shock and sequential organ failure assessment score were used for patient severity score assessments, assessed at the time of ECMO cannulation.^15^ Pre-ECMO hemodynamics were obtained from catheterization lab reports precannulation, and post-ECMO hemodynamics were recorded as an average of validated measurements by pulmonary artery catheterization during the 24-hour period postcannulation. AKI was defined according to the Kidney Disease Improving Global Outcomes 2012 guidelines. Major bleeding was defined as a bleed resulting in a hemoglobin drop of ≥3 g/dL, necessitating blood transfusion or requiring surgical or endovascular intervention. Thrombocytopenia was identified as a drop in platelets by ≥50% of baseline or to a nadir of <100,000/μL. Retroperitoneal bleeding in case of transcaval access was defined according to the modified Valve Academic Research Consortium definitions.^16^^,^^17^

### Statistical analysis

Continuous variables were described by median (IQR) and categorical variables by frequency rates and percentages. The Mann-Whitney *U* test or *t* test was used to compare continuous variables, whereas χ^2^ or Fisher exact tests were used to compare categorical variables. Paired univariate analysis was performed for comparison of pre–LAVA-ECMO and post–LAVA-ECMO hemodynamics, with a significance threshold of *P* < .05 on 2-sided alpha testing.

## Results

### Patient characteristics

**Figure 1:**
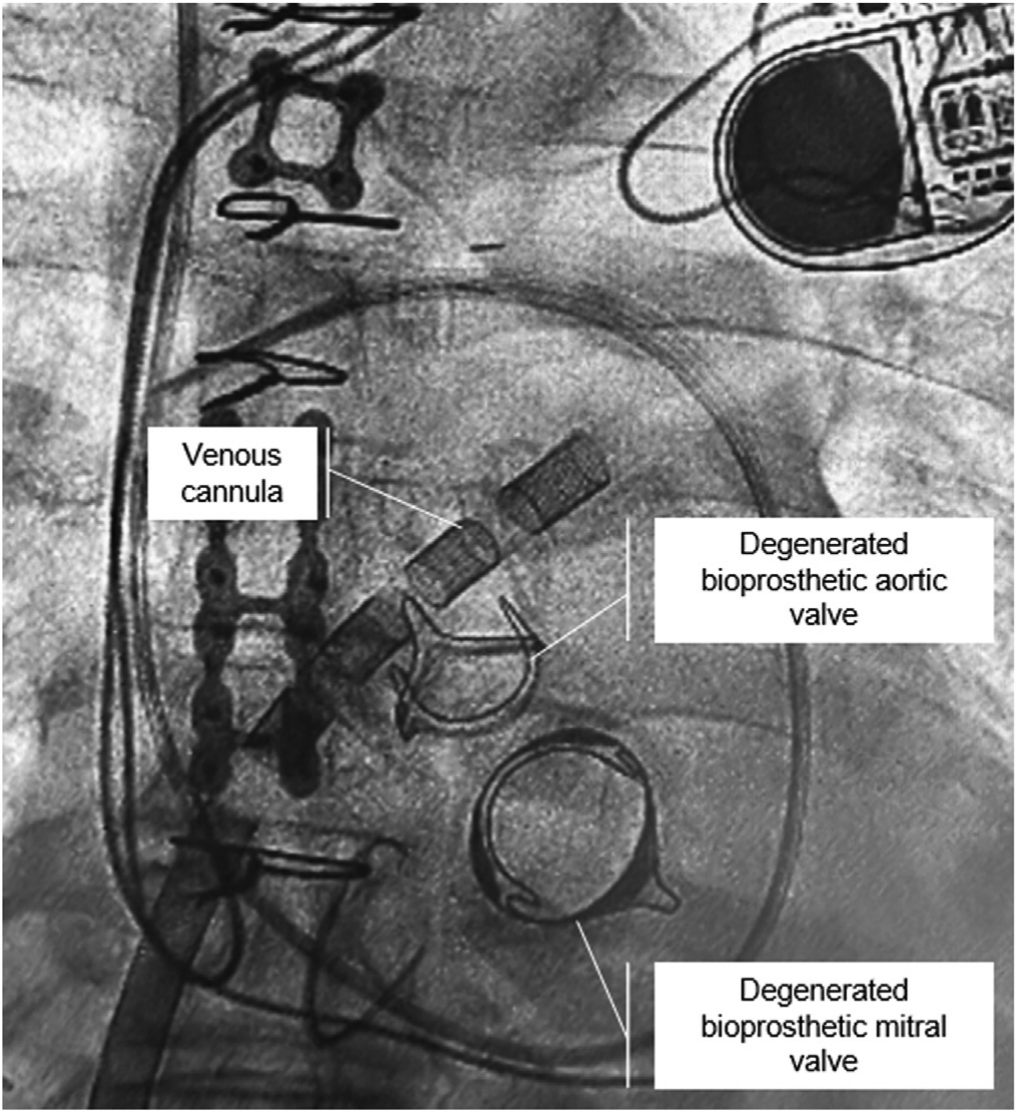
**Example of left atrial veno-arterial extracorporeal membrane oxygenation (LAVA-ECMO) cannulation in valvular cardiogenic shock.** The patient presented in valvular cardiogenic shock related to critical degenerative stenosis of the aortic and mitral bioprosthetic valves. The patient was first stabilized with LAVA-ECMO. The cannula is placed transeptally in the left atrium and ideally in the left upper pulmonary vein. The patient was then treated with simultaneous transcatheter aortic and mitral valve-in-valve while on LAVA-ECMO support at a later stage and discharged alive from the hospital.

During the study period, a total of 522 patients presenting with CS underwent VA-ECMO cannulation of whom 68 (13%) had LAVA-ECMO. Of these, 18 patients (18/68; 26.5%) had VCS. Baseline clinical characteristics are presented in Table 1. The median age was 65 years (IQR, 59-72), 66.7% were men and most were White (72%). Most of the patients were in SCAI shock stage D or E. Etiologies of VCS are illustrated in Figure 1. The most frequent etiologies of VCS were aortic regurgitation (33.3%), mitral regurgitation (33.3%), and aortic stenosis (22.2%). Of these, 22.2% had multivalvular disease of which combined aortic and mitral regurgitation were the most frequently encountered VHD lesions. Procedural characteristics are described in Table 2. Transeptal cannulation was predominantly performed using intracardiac echocardiography (77.8%), and the right femoral vein and artery were the primary access sites for outflow and inflow cannulation, respectively. Transcaval access for the arterial cannula was used in 27.8% of patients due to severe peripheral artery disease or inadequate peripheral artery size. Reperfusion sheaths were used in 61.1% of cases.

**Table 1:** Baseline demographics, past medical history, and severity of illness pre-ECMO

	N = 18
Age, y	65 (59-72)
Sex	
Male	12 (66.7)
Female	6 (33.3)
Race	
White	13 (72.2)
Black	4 (22.2)
Body mass index, kg/m^2^	28.7 (23.0-33.1)
Medical history	
Hypertension	14 (77.8)
Diabetes mellitus	7 (38.9)
Chronic kidney disease	10 (55.6)
End-stage renal disease	2 (11.1)
Coronary artery disease	7 (38.9)
Prior PCI	2 (11.1)
Prior CABG	4 (22.2)
Severity of illness	
Baseline lactate, mmol/L^a^	2.2 (1.7-3.0)
Baseline SAVE score^b^	–3.5 (–6.0 to 0.3)
Baseline SOFA score^b^	12.5 (10.3-14.8)
SCAI shock stage	
C	2 (11.1)
D	9 (50.0)
E	7 (38.9)

Values are median (IQR) or n (%).

CABG, coronary artery bypass graft; ECMO, extracorporeal membrane oxygenation; PCI, percutaneous intervention; SAVE, survival after veno-arterial extracorporeal membrane oxygenation; SCAI, Society for Cardiovascular Angiography & Interventions; SOFA, sequential organ failure assessment.

a Highest value pre-ECMO cannulation.

b Worst score pre-ECMO cannulation.

**Table 2:** ECMO indications, cannulation information, and decannulation strategy

	N = 18
Indication for ECMO^a^	
Aortic stenosis	4 (22.2)
Aortic regurgitation	6 (33.3)
Mitral stenosis	1 (5.6)
Mitral regurgitation	6 (33.3)
Tricuspid regurgitation	1 (5.6)
Multivalvular disease	4 (22.2)
Aortic and mitral regurgitation	2 (11.1)
Aortic and mitral stenosis	1 (5.6)
Aortic and tricuspid regurgitation	1 (5.6)
Transseptal puncture guidance	
Intracardiac echocardiography	14 (77.8)
Transesophageal echocardiography	4 (22.2)
Transcaval access	5 (27.8)
Venous cannula location	
Right femoral vein	13 (72.2)
Left femoral vein	5 (27.8)
Arterial cannula location	
Right femoral artery	13 (72.2)
Left femoral artery	5 (27.8)
Additional LV unloading	
Inotrope	9 (50.0)
IABP	0 (0.0)
Impella	2 (11.1)
Reperfusion sheath used	11 (61.1)
Decannulated successfully	12 (66.7)
ASD closed postdecannulation	5 (27.8)
Method of ASD closure	
Percutaneous septal occlude device	2 (11.1)
Surgical closure	3 (16.7)

Values are n (%).

**Figure 2:**
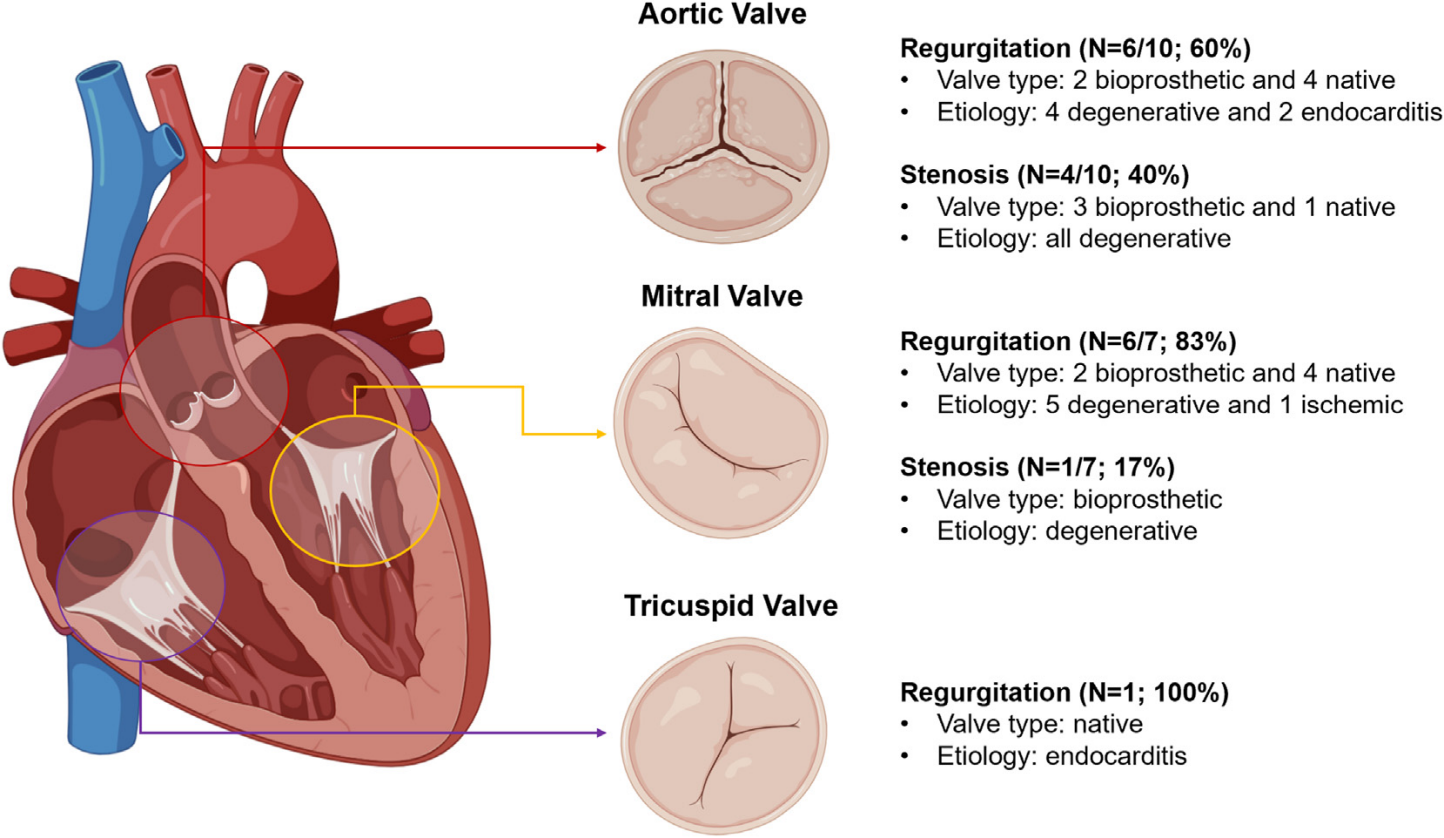
**Etiologies of valvular heart dysfunction–related cardiogenic shock undergoing left atrial veno-arterial extracorporeal membrane oxygenation cannulation**.

ASD, atrial septal defect; ECMO, extracorporeal membrane oxygenation; IABP, intra-aortic balloon bump.

a See Figure 2 for full details on the etiology and mechanism of valvular disease.

### Hemodynamic and clinical outcomes

**Figure 3:**
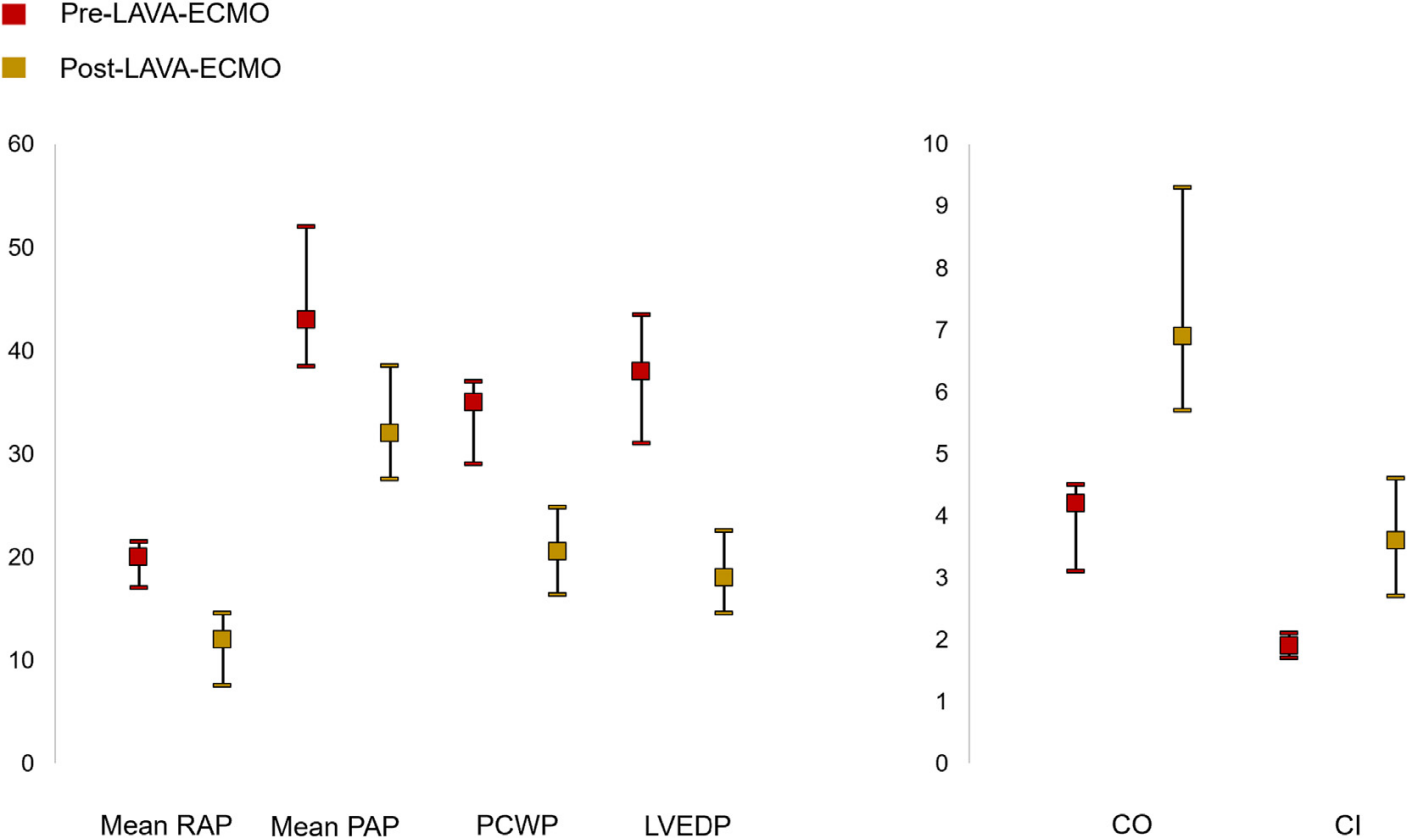
**Pre–LAVA-ECMO and post–LAVA-ECMO hemodynamics.** CI, cardiac index; CO, cardiac output; LAVA-ECMO, left atrial veno-arterial extracorporeal membrane oxygenation; LVEDP, left ventricular end-diastolic pressure; PAP, pulmonary artery pressure; PCWP, pulmonary capillary wedge pressure; RAP, right atrial pressure.

Invasive hemodynamics pre–LAVA-ECMO and post–LAVA-ECMO cannulation are presented in Table 3 and Figure 3. Overall, cannulation with LAVA-ECMO resulted in significant reductions in right atrial (RA) pressure (–8.0 mm Hg; 95% CI, –7.0 to –9.5; *P* = .004), pulmonary artery pressure (PAP; –11.0 mm Hg; 95% CI, –11.0 to –13.5; *P* = .039), PCWP (–14.5 mm Hg; 95% CI, –12.8 to –12.3; *P* < .001), and LVEDP (–20.0 mm Hg; 95% CI, –16.5 to –21.0; *P* < .013). The hemodynamic effects of LAVA-ECMO were consistent across different types of VHD lesions (Central Illustration). During treatment, 11.1% of patients required additional MCS implantation after LAVA-ECMO, with 1 patient receiving an Impella due to migration of the venous cannula in the right atrium. Clinical outcomes are summarized in Table 4. Survival to decannulation was 69.1%, and survival to hospital discharge was 44.4%. Complications included access-site bleeding (11.1%), vascular complications (11.1%), stroke (22.2%), and AKI (38.9%). There was 1 large retroperitoneal bleed in a patient who underwent transcaval cannulation due to extensive peripheral artery disease. There were no complications related to transeptal puncture, and there were no cases of pericardial effusion. The median time on ECMO was 5.5 days (IQR, 4.0-6.8). Survival to hospital discharge was 44.4%, with 27.8% undergoing atrial septostomy closure, either percutaneously or surgically during the procedure. Five patients (27.8%) underwent transcatheter aortic valve replacement, 3 (16.7%) underwent transcatheter mitral valve repair or replacement and 3 underwent surgical aortic or mitral valve replacement (16.7%). In-hospital mortality in patients who underwent a valve procedure (n = 11) was 27.3%. Among these patients, all but 1 patient survived until decannulation, whereas 2 patients died after decannulation.

**Table 3:** Preinvasive and postinvasive hemodynamics after LAVA-ECMO cannulation

	N^a^	Pre-ECMO	Post-ECMO	Δ (95% CI)	*P* value
Mean RA pressure, mm Hg	15	20.0 (17.0-21.5)	12.0 (7.5-14.5)	8.0 (7.0-9.5)	.004
PA pressure (systolic), mm Hg	15	65.0 (52.0-74.0)	45.0 (36.0-49.5)	18.5 (14.3-21.7)	.026
PA pressure (diastolic), mm Hg	15	34.0 (25.0-37.5)	21.0 (19.0-31.0)	12.9 (10.0-14.4)	.052
Mean PA pressure, mm Hg	15	43.0 (38.5-52.0)	32.0 (27.5-38.5)	11.0 (11.0-13.5)	.039
Mean PCW pressure, mm Hg	15	35.0 (29.0-37.0)	20.5 (16.3-24.8)	14.5 (12.8-12.3)	.003
LV end-diastolic pressure, mm Hg	7	38.0 (31.0-43.5)	18.0 (14.5-22.5)	20.0 (16.5-21.0)	.013
Cardiac output, L/min	15	4.2 (3.1-4.5)	6.9 (5.7-9.3)	3.9 (2.6-4.8)	<.001
Cardiac index, L/min/m^2^	15	1.9 (1.7-2.1)	3.6 (2.7-4.6)	1.9 (1.0-2.5)	<.001

Values are median (IQR).

ECMO, extracorporeal membrane oxygenation; LAVA-ECMO, left atrial veno-arterial extracorporeal membrane oxygenation; LV, left ventricular; PA, pulmonary artery; PCW, pulmonary capillary wedge; RA, right atrial.

a Indicates the number of patients with data available for each hemodynamic parameter.

**Central Illustration:**
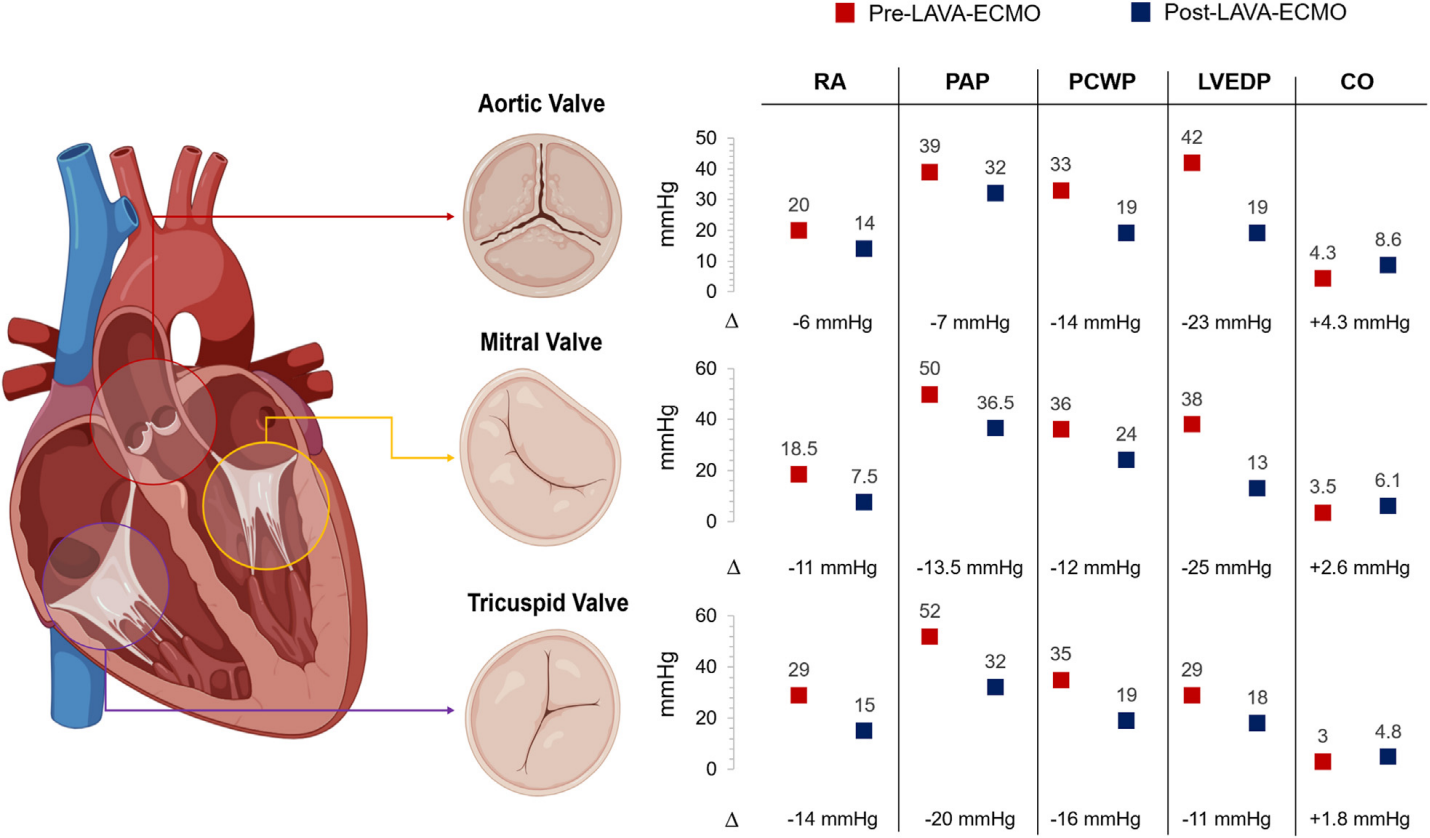
**Hemodynamic effects of left atrial veno-arterial extracorporeal membrane oxygenation (LAVA-ECMO) across types of valvular cardiogenic shock.** Numbers are median values in mm Hg pre–LAVA-ECMO (red dot) and post–LAVA-ECMO (blue dot) support. Full data are provided in Suppemental Table S1. CO, cardiac output; LVEDP, left ventricular end-diastolic pressure; PAP, pulmonary artery pressure; PCWP, pulmonary capillary wedge pressure; RA, right atrium.

**Table 4:** Complications and outcomes

	N = 18
Complications	
Return to the catheterization laboratory	4 (22.2)
Required additional MCS	2 (11.1)
Impella	1 (5.6)
VAV cannulation	1 (5.6)
Access site bleeding	2 (11.1)
Required blood transfusion	14 (77.8)
Arterial injury	2 (11.1)
Dissection	1 (5.6)
Pseudoaneurysm	0 (0.0)
Retroperitoneal bleed	2 (11.1)
Large	1 (5.6)
Limb ischemia	0 (0.0)
Infection	1 (5.6)
Stroke	4 (22.2)
Ischemic	1 (5.6)
Hemorrhagic	3 (16.7)
Acute kidney injury	7 (38.9)
Required RRT	3 (16.7)
Clinical outcomes	
Survival to transcatheter or surgical valve procedure	11 (61.1)
TAVR	4 (22.2)
TMVR	1 (5.6)
Valve-in-Valve TAVR	1 (5.6)
Valve-in-Valve TMVR	2 (11.1)
Surgical AVR	2 (11.1)
Surgical MVR	1 (5.6)
Survival after decannulation	11 (61.1)
Survival to hospital discharge	8 (44.4)
30-d survival from ECMO cannulation	8 (44.4)
Total days on ECMO, d	5.5 (4.0-6.8)

Values are median (IQR) or n (%).

AVR, aortic valve replacement; ECMO, extracorporeal membrane oxygenation; MCS, mechanical circulatory support; MVR, mitral valve replacement; RRT, renal replacement therapy; TAVR, transcatheter aortic valve replacement; TMVR, transcatheter mitral valve replacement/repair; VAV, veno-arterio-venous.

## Discussion

Valvular CS is associated with high morbidity and mortality and its treatment remains challenging. In fact, specific VHD lesions may contraindicate or make the use of certain MCS devices ineffective (eg, severe aortic regurgitation and IABP or Impella). By actively draining blood from the left and right atria, LAVA-ECMO is an MCS strategy that provides complete cardiocirculatory support and oxygenation while simultaneously providing biventricular unloading. Thus, its use may be particularly attractive in VCS. The present study is the largest series reported to date of LAVA-ECMO in VCS. The main findings of our study are as follows: (1) based on preinvasive and postinvasive hemodynamics, MCS with LAVA-ECMO resulted in complete cardiocirculatory support and active LV unloading reflected by the substantial reductions in LVEDP, PAP, and PCWP; (2) importantly, the hemodynamic effects of LAVA-ECMO were consistent across types of VCS (Central Illustration); (3) the procedure was technically safe, with no complications from transeptal puncture or venous ECMO cannula placement in the LA; rates of vascular and bleeding complications, as well as in-hospital mortality were in line with previous reports of VA-ECMO in VCS^2^; (4) within this setting, LAVA-ECMO served as a bridge to transcatheter or surgical interventions to address the underlying VHD lesions causing VCS.

Despite advances in MCS devices, CS remains associated with substantial morbidity and mortality.^15^ VCS accounts for 10% to 20% of CS etiologies and is associated with even higher risk of mortality compared with nonvalvular CS. In the setting of VCS, choices for MCS devices are limited and these patients have been excluded from randomized controlled trials evaluating MCS devices. Also, no guidelines exist on how to best manage patients in VCS. For example, severe aortic regurgitation contraindicates the use of IABP and Impella, as well as conventional VA-ECMO as it would result in a further increase in LVEDP and worsening pulmonary edema. LAVA-ECMO is a novel technique that consists of the transeptal placement of a fenestrated venous cannula to simultaneously drain blood from both atria and therefore provide biventricular unloading. As the blood flows directly from the LA and RA this strategy results in reductions in LVEDP, PCWP, and PAP, as opposed to conventional VA-ECMO. Therefore, it may be particularly beneficial in the case of VCS due to left-sided or right-sided VHD.

The use of LAVA-ECMO in the setting of VCS has so far been limited to single case reports or case series. In our study, LAVA-ECMO had consistent beneficial hemodynamic effects across types of VCS, with substantial reductions in LVEDP, PCWP, and PAP irrespective of the valve involved (Central Illustration). Of note, LAVA-ECMO served as a bridge to a transcatheter or surgical intervention for VHD in around 70% of the patients. Among patients who underwent a procedure to address the underlying VHD, we observed a relatively high in-hospital survival rate and all but one patient survived until ECMO decannulation. A treatment strategy of stabilizing patients presenting in VCS with LAVA-ECMO as a bridge to a transcatheter or surgical intervention warrants prospective investigation. LAVA-ECMO can provide ongoing hemodynamic support and enable high-risk transcatheter structural interventions particularly when directed to the aortic valve. When mitral valve interventions are necessary, the LAVA cannula can be withdrawn in the RA and the iatrogenic septal defect can be used to reaccess the LA.

Rates of adverse events in our study were acceptable when compared with traditional percutaneous VA-ECMO. Importantly, there were no complications related to transeptal puncture and LA cannulation with most of the cases performed using intracardiac echocardiographic guidance. As LAVA-ECMO provides active biventricular unloading, it obviates the need for a second MCS device for LV venting (eg, IABP or Impella) or RV support, therefore potentially reducing the risk of further vascular and bleeding complications related to an additional MCS device. This feature differentiates LAVA-ECMO also from LA-to-femoral artery bypass (eg, TandemHeart) where blood is selectively drained from the LA whereas, with LAVA-ECMO, blood is actively drained from both atria via a multistage venous cannula. In addition, LAVA-ECMO can provide greater hemodynamic support as well as oxygenation compared with the TandemHeart device.

### Limitations

Our study has several limitations that need to be disclosed. This is a retrospective single-center analysis, therefore subject to selection and treatment bias. The sample size was small, as it only included the group of patients with VHD-related CS from a larger CS cohort. The decision for the use of MCS was determined by the treatment team and the study lacks a conventional VA-ECMO control group. LAVA-ECMO requires expertise with a transeptal puncture, intracardiac echocardiography, and management of large-bore access; therefore, its generalizability to less experienced centers may be limited. In addition, dedicated cannulas to perform LAVA-ECMO are not available. The clinical decision-making to pursue LAVA-ECMO cannulation, rather than conventional VA-ECMO was not collected nor was it defined a priori based on a standardized protocol. Finally, the sample size of the present report is small and we are unable to assess the effects of LAVA-ECMO according to specific hemodynamic profiles. Further research to identify patients who may benefit from LAVA-ECMO versus conventional VA-ECMO is necessary.

## Conclusion

In patients with CS, related to VHD, LAVA-ECMO, a strategy of complete cardiocirculatory support with direct LA drainage and active LV unloading, was feasible and associated with favorable hemodynamic effects across different types of VCS. Patients on LAVA-ECMO who underwent a transcatheter or surgical valve procedure had relatively high survival rates. LAVA-ECMO may serve as a bridge treatment strategy to structural or surgical interventions in patients with VCS.

## Supplementary material

Suppemental Table S1
