# Supplementary material for: Left Atrial Veno-Arterial Extracorporeal Membrane Oxygenation In Valvular Cardiogenic Shock
Source: J Soc Cardiovasc Angiogr Interv. 2025 May 1;4(5):102615. doi: 10.1016/j.jscai.2025.102615 (PMC12126062; doi:10.1016/j.jscai.2025.102615)
Supplement: Suppemental Table S1 [file mmc1.docx]

**Supplemental Table S1.**

|  |  | **Aortic** | | **Mitral** | | **Tricuspid** | |
| --- | --- | --- | --- | --- | --- | --- | --- |
|  |  | Pre- | Post- | Pre- | Post- | Pre- | Post- |
| RA | Median | 20 | 14 | 18.5 | 7.5 | 29 | 15 |
|  | q25 | 17 | 10 | 17.3 | 6 | - | - |
|  | q75 | 22 | 15 | 19.8 | 12.8 | - | - |
| PA | Median | 39 | 32 | 50 | 36.5 | 52 | 32 |
|  | q25 | 38 | 28 | 44.3 | 22.3 | - | - |
|  | q75 | 47 | 33 | 52.8 | 42.5 | - | - |
| PCWP | Median | 33 | 19 | 36 | 24 | 35 | 19 |
|  | q25 | 21 | 16 | 35.3 | 17 | - | - |
|  | q75 | 35 | 22 | 38.3 | 30 | - | - |
| LVEDP | Median | 42 | 19 | 38 | 13 | 29 | 18 |
|  | q25 | 32 | 18 | 34 | 10 | - | - |
|  | q75 | 45 | 26 | 40 | 14.5 | - | - |
| CO | Median | 4.3 | 8.6 | 3.5 | 6.1 | 3.0 | 4.8 |
|  | q25 | 4.2 | 5.7 | 3.1 | 5.7 | - | - |
|  | q75 | 4.5 | 9.3 | 3.9 | 8.6 | - | - |
| CI | Median | 1.9 | 3.6 | 1.8 | 3.6 | 1.3 | 2.1 |
|  | q25 | 1.6 | 3.2 | 1.7 | 2.6 | - | - |
|  | q75 | 2.0 | 4.4 | 2.1 | 4.8 | - | - |

Results are displayed as median and interquartile range with pre- or post-LAVA-ECMO. CI = Cardiac Index; CO = Cardiac Output; LVEDP = Left Ventricular End-Diastolic Pressure; PA = Pulmonary Artery; PCWP = Pulmonary Capillary Wedge Pressure; RA = Right Atrium.
